# Phage Therapy Is Partially Successful in Rescuing Hornworms from *Pseudomonas* Infections

**DOI:** 10.3390/antibiotics15080757

**Published:** 2026-08-06

**Authors:** Jennifer L. Wilcox, Emma K. Spencer, Tracey L. Peters, Craig R. Miller, James J. Bull

**Affiliations:** 1 Department of Biological Sciences, University of Idaho, Moscow, ID 83844, USAcrmiller@uidaho.edu (C.R.M.); jbull@uidaho.edu (J.J.B.); 2 Department of Animal, Veterinary and Food Sciences, University of Idaho, Moscow, ID 83844, USA; tpeters@uidaho.edu; 3 Institute for Modeling Collaboration and Innovation, University of Idaho, Moscow, ID 83844, USA

**Keywords:** bacteriophage, *Manduca sexta*, tobacco hornworm, animal models

## Abstract

Background: Phage therapy is one of the few alternatives to confront the tide of drug-resistant bacteria. Yet phage therapy is often applied knowing little more than that the therapeutic phages can grow on the infecting bacterium. The lack of convenient, inexpensive infection models slows progress in discovering principles that may improve phage therapy success. Here we explore hornworms, the larvae of hawkmoths, as a new model host for phage therapy. Methods: We injected fifth instar hornworms with *Pseudomonas aeruginosa* and, in some, also simultaneously administered a single dose of one of three phages. Results: Phage treatment significantly improved survival relative to bacteria-only controls, although only modestly. Phages did not significantly affect hornworm weight gain among survivors. Differences in survival and weight among the three phage treatments were not statistically significant despite large genomic differences among the phages. Conclusions: Comparing hornworms to the more commonly used waxworms, hornworms, offers additional phenotypes that can be easily assayed, but their need to be housed individually increases the effort to provide large numbers.

## 1. Introduction

Antibiotic-resistant bacteria pose a major risk to human health and are estimated to kill over a million people per year globally [1]. The use of bacteriophages to treat bacterial infections, phage therapy, has shown promise as a sometimes viable option when traditional antibiotics fail [2,3,4]. However, phage therapy in the US is currently restricted to companionate care, making large-scale human trials challenging. In vitro studies can easily measure various properties of phage efficacy, but it is not known how well in vitro characteristics of phage efficacy transfer to in vivo environments [5]. Animal models provide a benefit here, bridging that knowledge gap. Mouse model systems have been standard for many years, but in the last decade, invertebrate models such as waxworms have become increasingly popular [6,7]. One challenge with the waxworm model is its small size, allowing only binary measurements of mortality without the inclusion of more continuous morbidity factors such as weight over multiple days of observation. Here we use a new, substantially larger invertebrate system in testing phage therapy: hornworms, or larvae of the *Manduca sexta*, the six spotted hawkmoth. We use hornworms as a host to test the efficacy of phages in treating artificial, acute infections of *Pseudomonas aeruginosa*. Our study follows a recent analysis of hornworms as a model of *Pseudomonas* infection and treatment with antibiotics [8,9] as well as building on previous studies utilizing hornworms as disease models and investigations on the effects of infection on hornworm physiology [10,11,12,13].

## 2. Results

**Figure 1:**
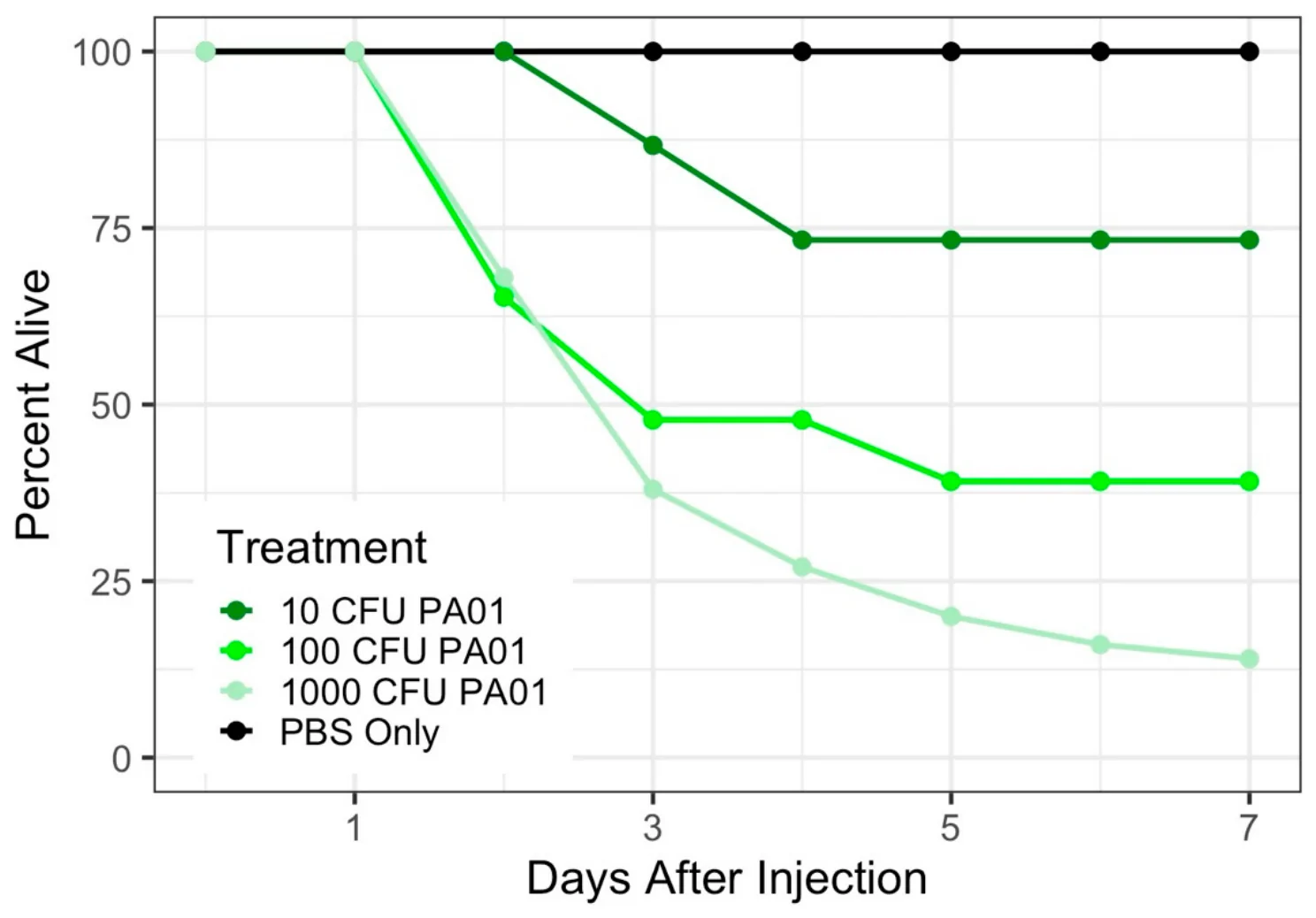
Time course of survival for hornworms injected with phosphate-buffered saline (PBS only) or PAO1 *P. aeruginosa* in 10 μL, with doses ranging from 10 to 1000 CFU. Survival rates decrease as bacterial doses increase, spanning 17.8–73.3% survival. Our study of the effects of phage was limited to a dose of approximately 10^3^ bacteria, as approximated by the lowest curve on the graph. All worms counted as survivors entered pupation by at least day 7 but may have entered pupation before day 7. Data from Table 1. From top to bottom on day 7: [No. survivors, no. dead (credible interval for probability of survival)] are [9, 0 (0.70–1.0), [11, 4 (0.48, 0.89)], [8, 16 (0.18–0.53)], [11, 43 (0.12, 0.33)].

**Figure 2:**
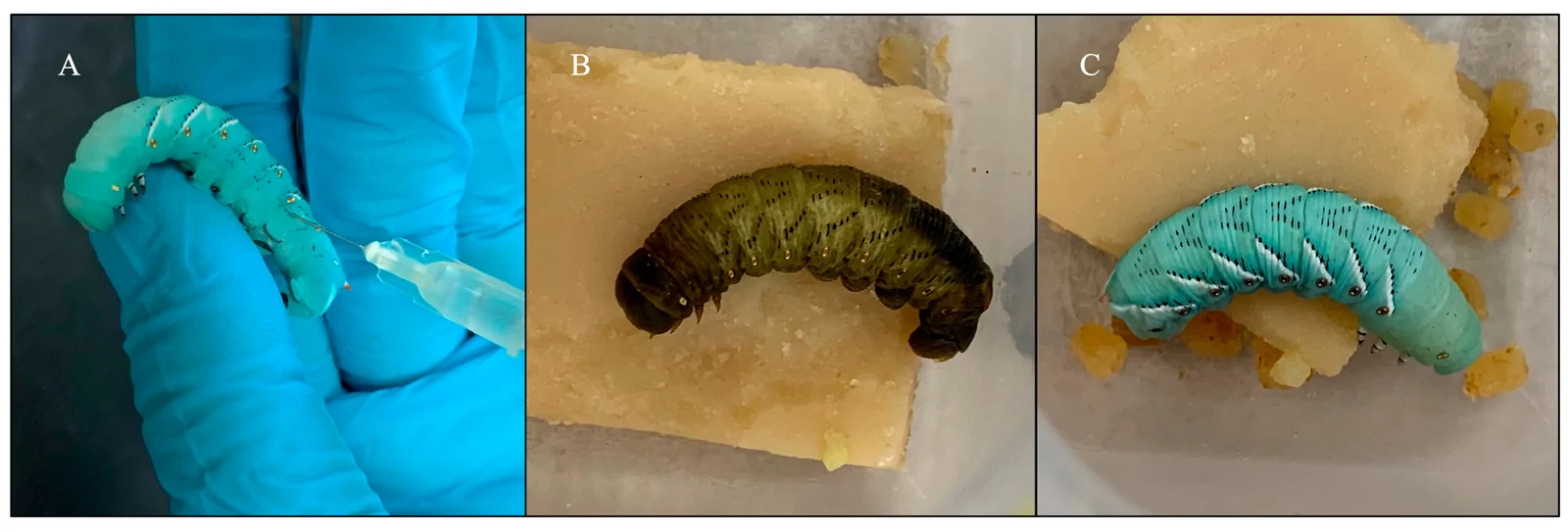
Fifth instar hornworms. (**A**) A snapshot during a hornworm injection. Injections were administered on the lower side between the third and fourth prolegs. Both bacterial cells and phages were injected in 10 μL doses separately. Phage injections were administered on the opposite side from bacterial injections. (**B**,**C**) show dead hornworms on day 2 post-injection. (**B**) is that of a worm receiving only bacterial cells, while the worm in panel (**C**) was also injected with phages. The melanism shown in (**B**) is typical of dead larvae after several hours; panel (**C**) shows a recent death.

*Manduca sexta* larvae (tobacco hornworms) were reared from eggs using standardized lab protocols [8] until they entered their fifth and last instar, at which time they were injected with a PAO1 strain of *Pseudomonas aeruginosa*. We sought a bacterial dose that caused substantial long-term mortality but was not so severely and rapidly lethal as to preclude the possibility of treatment success. Injections of PAO1 in concentrations ranging from 10^1^ to 10^3^ CFU reveal that worm survival rates correlated with bacterial dose administered (Figure 1 and Figure 2). We used a bacterial dose of approximately 5 × 10^3^, as it satisfied our objectives (for ease of notation, we refer to this dose as 10^3^).

At the same time as PAO1 injections, some worms were also injected with 10^6^–10^9^ of one of three phages pre-determined to target PAO1 (*Pseudomonas* phage Φ176 and two environmental isolates, Table 1). Genomic analysis revealed phages to be members of different viral families: *Luzseptimavirus* (SI_6), *Pbunavirus* (SI_7), and *Litunavirus* (phage Φ176). Our phage Φ176 shared 99.1% identity to the published Φ176 genome (NCBI accession KM411960.1), with a 662 bp deletion in a hypothetical protein (LQZNTSYL_CDS_0088), 7 single-base deletions in homopolymeric tracts (plausibly the result of sequencing and assembly methodology using Oxford nanopore technology), and a two-base insertion in the same hypothetical protein as the 662 bp deletion. Phages isolated from sewage, SI_6 and SI_7, represent two new phage species from the genera *Luzseptimavirus* and *Pbunavirus,* respectively. SI 6 showed 92.9% identity to *Luzseptimavirus* KPP21 (LC064302.1). SI_7 showed 93.1% identity to *Pbunavirus* Epa61 (MK317959.1).

Beyond genomics, no further characterization of these phages was carried out, as is commonplace with phage therapy using wild phages [3].

### 2.1. Expected Killing from Phage Treatment

We are interested if phages rescue worms from the infection by killing bacteria. The simplest possible treatment is a single dose of phage administered at the time of bacterial inoculation, which is what we used. To set expectations for the possible magnitude of phage killing of bacteria in the short term, we seek insight from standard modeling approaches. In liquid under mass action dynamics, bacterial survival amid phage attack is given by the following:

B’ = −k·P·B,(1)

where B is the density of uninfected bacteria (function of time), P the density of free phage (function of time), k the adsorption rate constant, and B’ the per-minute change in uninfected bacteria [14,15]. A second equation governs phage dynamics but given the vast excess of phage over bacteria in our inoculations, a suitable approximation for short-term dynamics is to ignore changes in phage density. From Equation (1), the density of uninfected bacteria follows:

B(t) = e^−kPt^, (2)

assuming no change in phage density (which will be approximately correct in the short term, given the large excess of phage over bacteria). In vivo infection parameters are necessarily unknown, but if we consider the adsorption rate constant to be in the range of 10^−9^ to 10^−10^ mL/min (10^−9^ is typical for many phages in liquid, 10^−10^ is low) and the liquid volume of the larva to be 1 mL (suitable for early fifth instar), the fraction of infected bacteria killed in the first hour can be high or low, depending on phage input and the adsorption rate constant (Table 2), assuming mass action dynamics inside the worm.

Beyond the short term, phage will be lost to decay/death but will increase from infections being converted to phage progeny. With low bacterial density and even a modest decay rate, phage may fail to increase their numbers despite ongoing infection (a concept known as the “proliferation threshold” applies here [16]). With low bacterial densities, any phage amplification is necessarily small simply because phage progeny is limited by the number of infections, thus further justifying the approximation in Equations (1) and (2).

The main unknown in applying these calculations is the internal flow of bacteria and phage within the worm. If bacteria and phage remain suspended in hemolymph, the mass action assumptions may provide a suitable approximation. Instead, bacteria collect and concentrate at limited sites, then the mass action assumption does not apply, and local phage amplification can spread to nearby bacteria at the same site, creating a much more effective killing than suggested by the mass action equations. The equations merely provide a foundation on which to help understand the system.

### 2.2. All Phage Treatments Increased Larval Survival Rates

After injection with bacteria (and possibly phage; Table 1), worms were monitored and weighed daily and recorded as deceased when tactile probing failed to elicit a response. Recording continued until all worms died or entered pupation (up to 9 days post-injection). Worms surviving to the pre-pupal stage were counted as “alive” throughout the duration of the experiment, but weight was no longer recorded after the onset of pupation.

**Figure 3:**
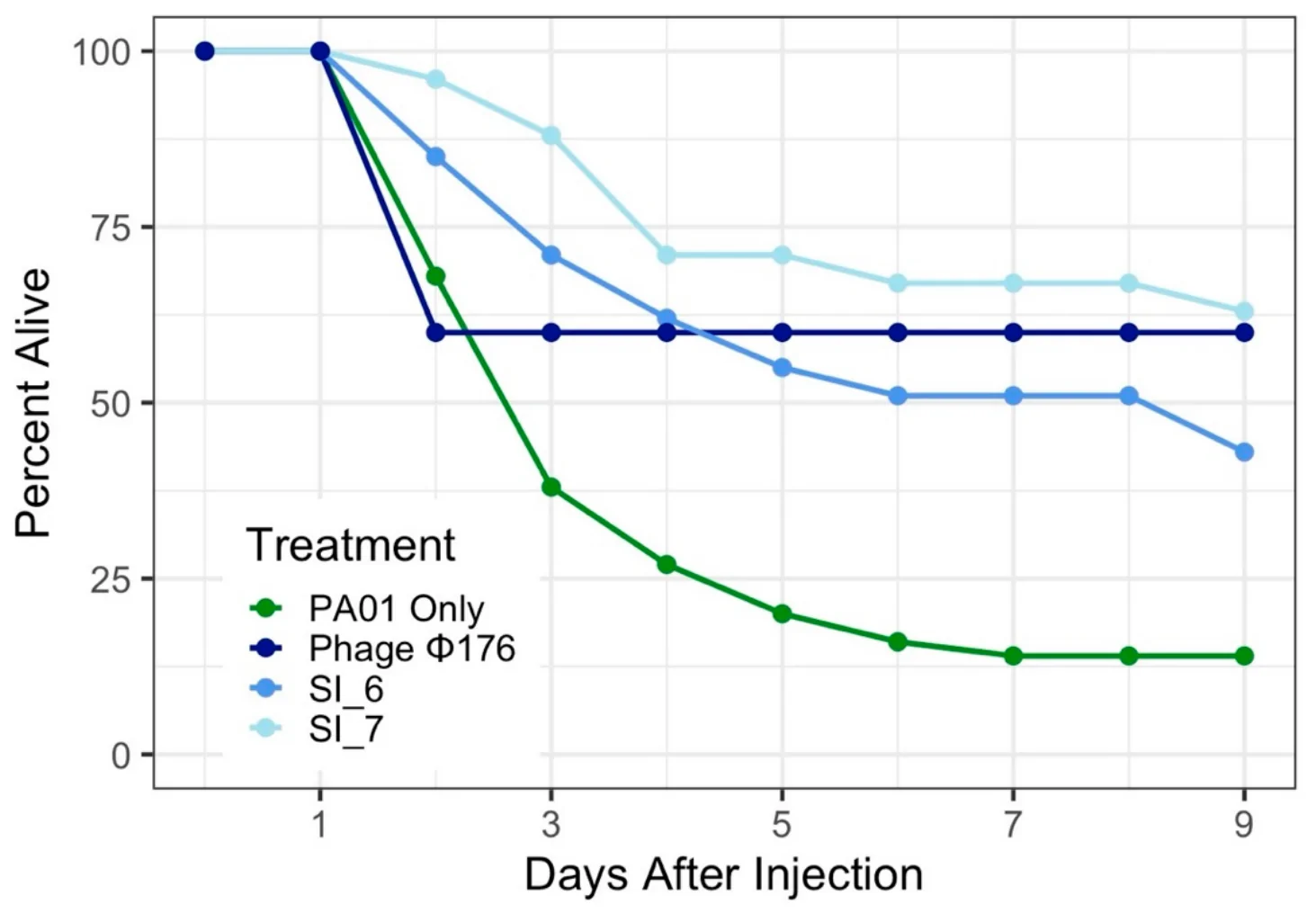
Time course of survival by treatment for all worms receiving PAO1 doses of 10^3^ CFU. Survivors at day 9 include all individuals who lived to enter pupation, which may have occurred before day 9. Survival at day 9 was significantly heterogeneous among the four groups (X^2^(3) = 16.2, *p* < 0.001), and the difference in survival between the bacteria-only treatment and all-phages combined remained highly significant. With the bacteria-only treatment removed, the differences among individual phages were not significant (X^2^ = 3.5258, *p* = 0.17). Data from Table 1; all SI_6 doses were combined into one treatment group for these tests and for the curve shown. From top to bottom on day 9: [No. survivors, no. dead (credible interval for probability of survival)]: [15, 9 (0.43–0.80)], [6, 4 (0.30–0.88)], [40, 53 (0.33–0.53)], [8, 37 (0.09, 0.32)].

Treatment with all three phages improved larval survival compared to phage-free controls. Survival rates for worms receiving phage treatment were between 43.5% and 62.5% (all doses for SI_6 were combined, as there was no significant heterogeneity among the four experiments), while the survival rate for those injected with PAO1 only was 17.8% (Figure 3). A Chi-squared test of surviving versus dead worms on day 9 (2 × 4 table) confirmed significant differences between PAO1-only controls and the three phage treatments (X^2^(3) = 16.2, *p* < 0.001). Survival differences between different phage treatments, however, were not significant (X^2^(2) = 3.5258, *p* = 0.17), and the survival rate across all phages combined was 47%. Survival was assessed only up to the onset of pupation. Outcomes beyond this point—including the transition to the pupal stage and subsequent development—were not evaluated. The potential effects of infection on survival during and after pupation are briefly discussed below.

### 2.3. No Significant Growth Differences Among Treatments

**Figure 4:**
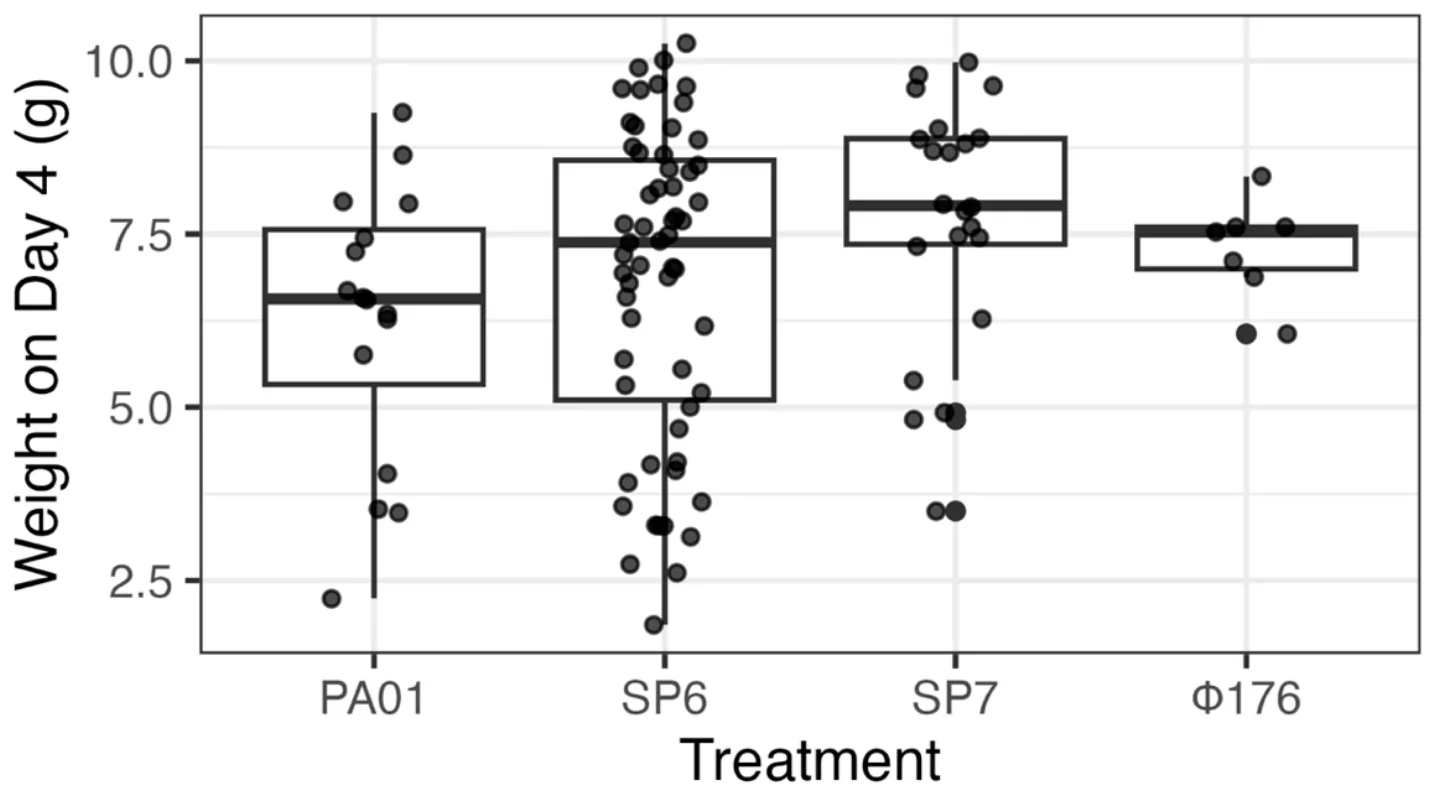
Worm weight on day 4. Mid-experiment weights of all surviving worms were measured to compare treatments. All doses of SI_6 have been combined into one treatment. By ANOVA, overall differences were not significant. F(3,100) = 1.87; *p* = 0.14.

**Figure 5:**
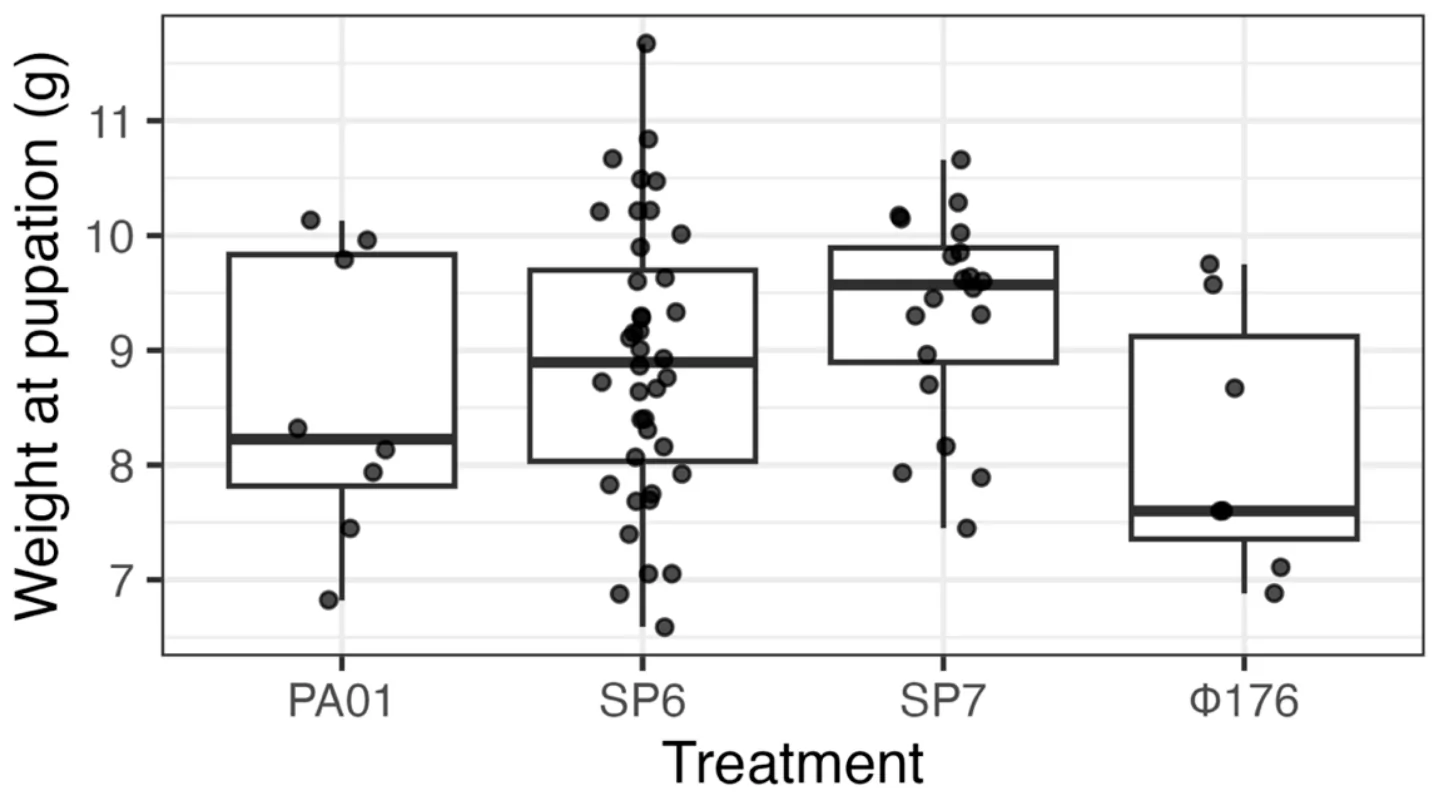
Worm weight at entry into pupation. Final larval weight was compared between phage treatments. By ANOVA, differences across treatments were not significant. F(3,71) = 2.176; *p* = 0.0983.

Uninfected larvae increase weight approximately 10-fold during the fifth instar, from ~1 to 10 gm. Worms were injected within a day following the onset of the fifth instar. Because of the immense growth of hornworms, we might expect that worm weight following injection will reflect differences in worm “health” and thus provide additional insight to phage efficacy not reflected by mortality. This view is further supported by weight differences observed among worms given different doses of PAO1 [9]. Worms in our study were weighed daily until death or entry into pupation. Weights at day 4 post-injection and at entry into pupation were compared across the three phage-injected groups and the bacteria-only group (Figure 4 and Figure 5). No significant heterogeneity was observed at either time, suggesting that weight was not an especially sensitive indicator of health or that worm weight/health was not intrinsically linked to treatment. The higher means of all three treated worm groups might justify repeating the study with larger samples, but even with the current group sizes, the scatter observed within groups shows that a major source of size variation comes from unmeasured effects.

### 2.4. Phage Inoculum Dose Does Not Predict Survival

**Figure 6:**
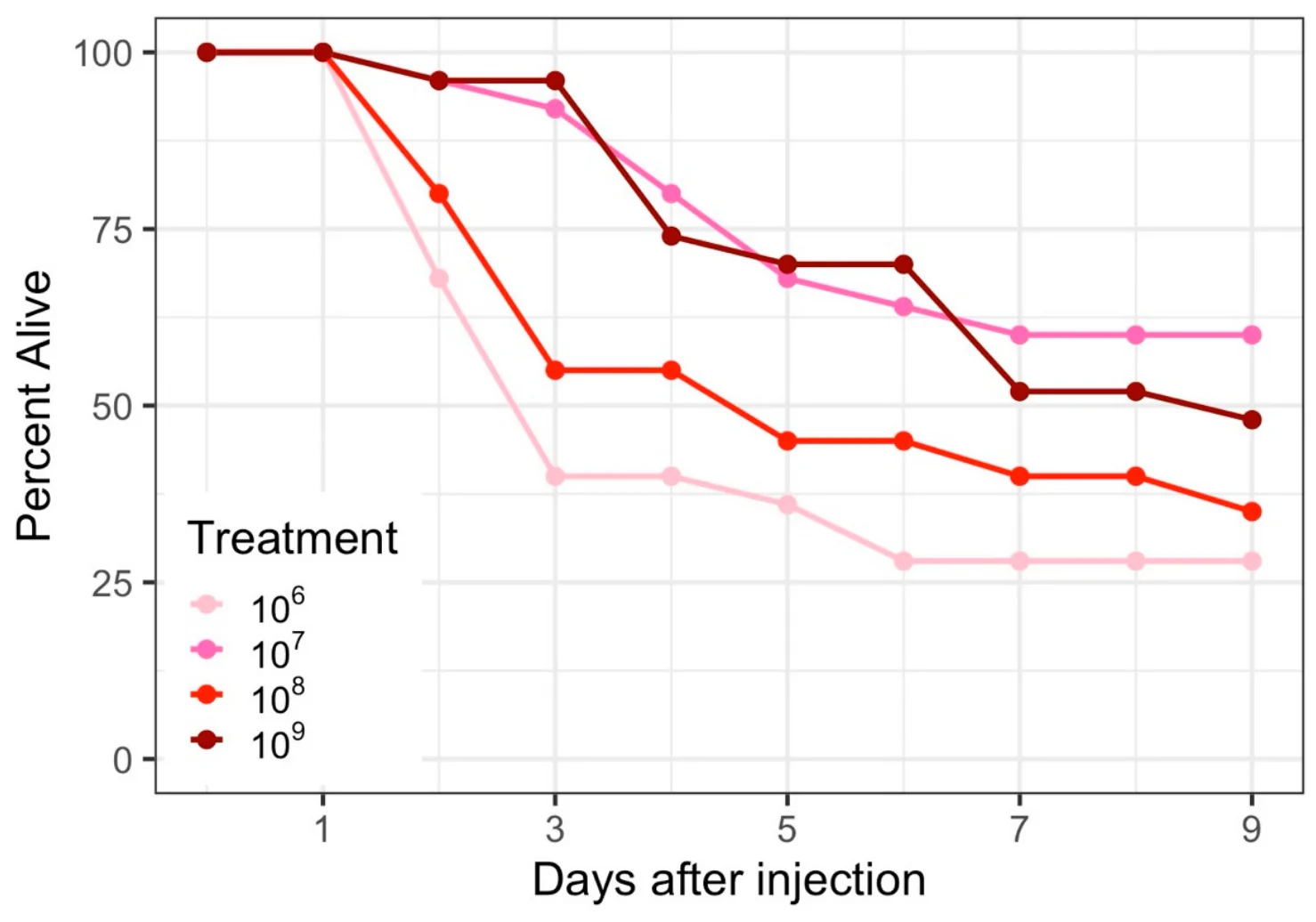
Survival comparisons of different doses of the same phage. SI_6 phage was administered in four different doses. Survival rates do not correlate to phage dose. In addition, a Chi-squared test failed to detect significant differences between doses (X^2^ = 2.1794, *p* = 0.54). Worms counted as survivors may have entered pupation before day 9. Data are provided in Table 1. From top to bottom on day 9: [No. survivors, no. dead (credible interval for probability of survival)]: [15, 10 (0.41–0.77)], [11, 12 (0.29–0.67)], [7, 13 (0.17–0.57)], [7, 18 (0.13, 0.47)].

We also investigated if phage dose influenced worm survival, as expected if bacterial killing directly translates into worm survival (recalling Equation (2)). Dose would not be expected to affect survival if phage rapidly amplify their numbers in the host, but as noted above, phages do not appreciably amplify at low bacterial densities (under mass action), in which case the inoculum size directly affects killing (Equation (2) and [17]). Day 9 survival was assessed when varying SI_6 dose by three orders of magnitude (Table 1, Figure 6). Although observed survival differed between treatments, the rank order of survival did not strictly follow phage dose (survival at 10^7^ is higher at 10^8^ and 10^9^), and a non-parametric rank test is not significant (*p* < 0.33). Furthermore, the Chi-squared test of heterogeneity across the four groups was not significant (X^2^(3) = 2.1794, *p* = 0.54). Likewise, when evaluating the relationship between log phage dose and larval survival for all phage treatments in Table 1, the correlation is only 0.28. If such effects of dose exist, they are overwhelmed by sampling error in our experiment.

The three phages included in this study were not all administered in equal doses (Table 1). We thus also considered whether differences exist between different phages of equivalent doses. Twenty-five worms in the SI_6 treatment group and all 10 worms of the phage Φ176 treatment group were injected with 4 × 10^7^ and 5 × 10^7^ phages, respectively. Likewise, all 24 worms in the SI_7 treatment group and 20 worms from the SI_6 treatment group were injected with approximately 10^8^ phages (Table 1). However, when comparing different phages at similar doses, we found no statistically significant differences in survival outcomes.

### 2.5. Surviving Pupae Treated with Phage Lack Detectible Pseudomonas

Assays so far have been confined to worm phenotypes. It is of equal interest to know whether phages are affecting bacterial loads, especially since worm phenotypes are affected by bacterial components even in the absence of live bacteria [9]. It is thus possible that phages are highly effective at eliminating bacteria (in conjunction with the worm’s immune system), even though worm phenotypes suggest lasting effects of the bacteria. The broader relevance of such a result would be that phages could be even more effective than suggested by survival. Although recent reviews of safety and toxicity of phages have found phages are generally well-tolerated both in animal models and humans [18,19], there is potential for adverse reactions both to toxins produced during phage lysis of bacterial cells as well as to bacterial components present in phage preparations. In one study using experimental infections of mice with high doses of bacteria, treatment by a lytic phage more commonly killed the mice than treatment with a lysis-defective phage that killed the bacteria without lysing [20]. A newer study showed that expression of PhoA from the phage genome, which can neutralize endotoxin during cell lysis, mitigated inflammatory responses during T7 phage treatment in both zebrafish and waxworms [21]. In view of the fact that injection with dead PAO1 and that injection of bacteria-free supernatant kills hornworms [9], it seems possible or even likely that immune reactions to endotoxin or other bacterial components alone may be contributing to the phage-treated hornworm deaths we observed.

**Figure 7:**
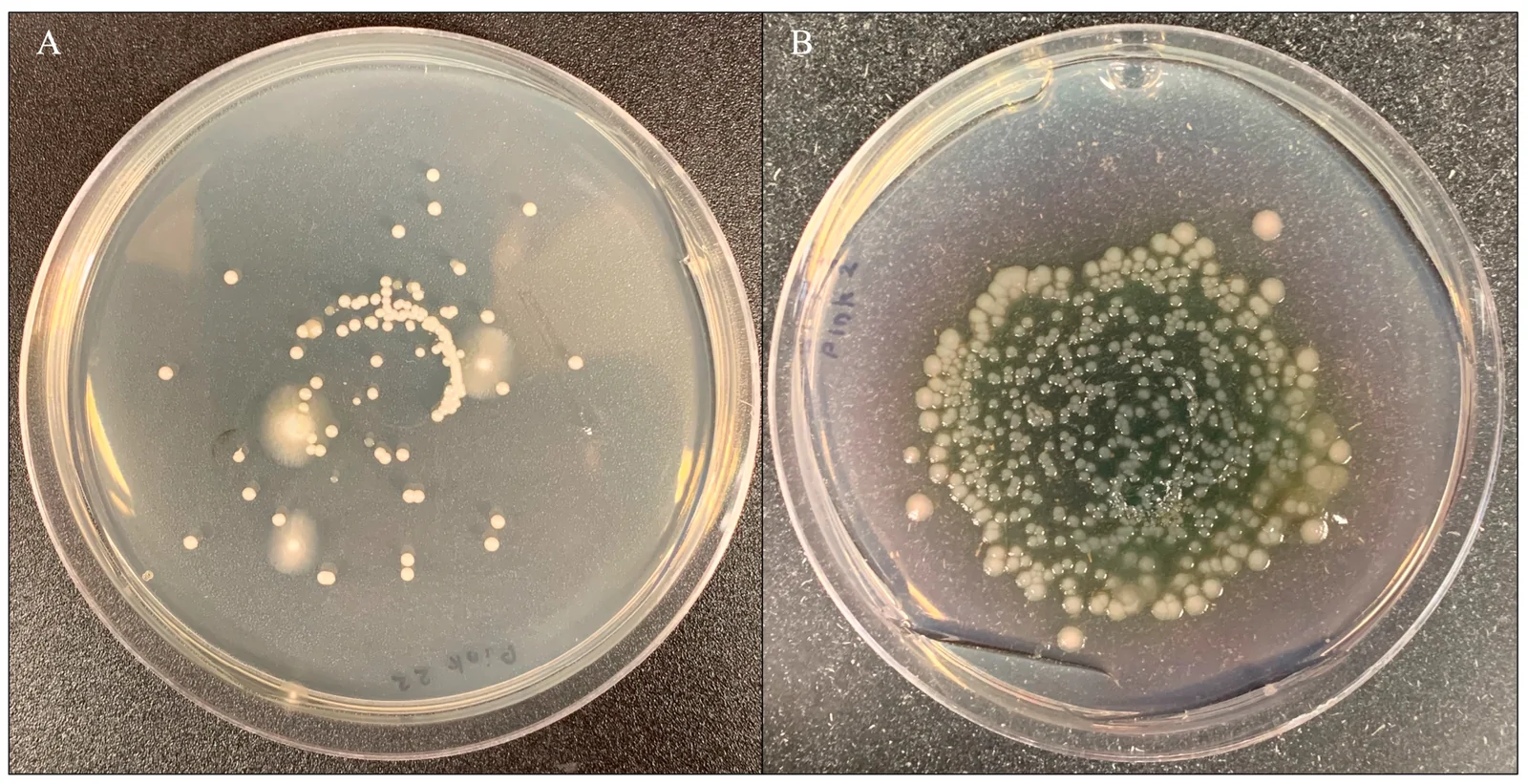
Contents of dissected pupae were plated on *Pseudomonas* identification plates to access CFU counts of *P. aeruginosa* 21 days post-injection. A total of 10 μL of pupal contents were plated for each sample. *Pseudomonas* identification media was used for plates, which turn green for quick identification of *Pseudomonas* species. (**A**) shows bacterial colonies from a phage-treated worm. Several different bacteria/fungi are present, but none were confirmed to be *Pseudomonas*. (**B**) shows bacteria from a PAO1 control worm with over 400 *Pseudomonas* colonies.

At the completion of the larval work, we became interested in whether PAO1 persisted in the pupal stage. One reason to think that PAO1 persists is that pupae from worms receiving bacterial injections were rarely observed to eclose, even after months of maintenance, despite evidence that the pupae were still alive (the normal time to eclosion is 2–5 weeks). To assess bacteria in pupae, we assessed small numbers from some of the studies reported above: three pupae from PAO1-only controls and six phage-treated pupae (Methods). A small portion of their contents was plated to measure *Pseudomonas* CFUs (Table 3, Figure 7); the small sample for controls stems from their low survival to pupation. Using *Pseudomonas*-identification plates, two of the PAO1-only control worms were found to have roughly 5 × 10^4^ *Pseudomonas* CFUs/mL. No PAO1 was detected in one of the PAO1-only controls nor in all six phage-treated worms, although other bacteria and fungi were present, possibly as surface contaminants.

These data are effectively anecdotal, as there was no attempt to standardize the timing of analysis. However, they at least raise the possibility that phages help in formally clearing the bacteria from the worm.

## 3. Discussion

We compared three different phages in the treatment of artificial *Pseudomonas aeruginosa* infections of a moth larva. This common nosocomial pathogen is often associated with burn wounds and cystic fibrosis patients and is a common target for phage therapy [22,23,24]. Many previous studies have utilized animal models to investigate efficacy of phages against *P. aeruginosa*, including mouse models [25,26,27,28] and invertebrate models such as waxworms (*Galleria mellonella*) [29,30]. Our study used a new insect model—hornworms (fifth instar larvae of the six-spotted hawkmoth, *Manduca sexta*).

Hornworms were injected with approximately 10^3^ CFUs of PAO1 *P. aeruginosa*, a dose from which only ~20% survived if untreated. Some larvae were also treated with a single inoculum of one of three phages in doses ranging from 10^6^ to 10^9^ PFU. Phage treatments significantly increased survival, but only to 47% overall; there were no significant differences between phages. Worm weights were recorded daily, and comparative analysis on day 4 post-injection and on entry into pupation showed no significant differences between treatments (with or without phages) despite the significant differences in survival. For one phage, we also investigated effects of phage inoculum dose on survival, with no evidence of an effect. A lack of a dose effect is not necessarily surprising, as the lowest dose (10^6^) may have been sufficient to kill enough bacteria to achieve maximum benefit. Pupal cocoons from surviving infected larvae were tested for the presence of *Pseudomonas*. Two of the three bacteria-only control pupae had approximately 10^4^ CFU, while the remaining control pupa and all six phage-treated pupae tested negative. This hints phage treatment may facilitate bacterial clearance (in conjunction with the immune system) if the worm survives, but a larger sample is obviously desired.

The seemingly modest rescue provided by phages seems puzzling at first. However, injection of killed *Pseudomonas* or of sterile supernatants of *Pseudomonas* cultures is fatal to a modest fraction of worms [9]. As the release of toxins accompanying phage lysis is unavoidable, some lethality is expected from this effect even if phages kill all bacteria [31,32].

For comparison to the recovery enabled by phages, a previous study evaluated *Pseudomonas* infections of hornworms when treated with antibiotics. In that work, the untreated infections survived at 6%, whereas the antibiotic-treated worms survived at 60% (cefepime) and 71% (gentamycin); the drugs were applied twice daily at approximately MIC for cefepime and ¼ MIC for gentamycin [8]. In contrast to the results here, antibiotic-treated worms grew significantly better than untreated worms but worse than worms not given bacteria. Thus, the phage treatment used here was somewhat inferior to the drug treatment in the previous study for survival, but the results are not easily comparable because only a single phage dose was administered. Nonetheless, the combination of both studies reveals a wide range of possible outcomes when using hornworms as a bacterial infection model.

Survival beyond entry into pupation was not formally monitored, but it appears to be affected by the infection. Duration of the pupal stage typically lasts 2–5 weeks [8], whereas our study observed some PAO1-injected pupae exceeding 6 months—and we observed eclosion in few of the approximately 80 individuals descended from treated larvae, with most remaining as pupae. Thus, it seems that prior infection with *Pseudomonas* is sufficient to delay or block the final stage of the life cycle, even if the pupa is not formally dead.

Despite expectations that the extensive growth of (fifth instar) hornworms would enable fine-scale assessment of treatment efficacy, the average weights of surviving worms showed no statistical association with average survival of the treatment. Although a larger sample might ultimately show an association with averages, the current data reveal that any such association is likely to be at best modest in magnitude and that a large between-individual variation in growth is the dominant property. The absence of such an association of mean weights with survival is compatible with infections obeying an all-or-none property—of being controlled or not—rather than spanning a gradation of intermediates. Perhaps survival of an infected hornworm indicates a type of normal physiology, to the point at which infection overwhelms the worm, whence the worm dies quickly.

One obvious next step is to monitor bacterial densities in the presence and absence of phages. Here that was done only for a small sample of pupae; ideally, we would like to know how quickly phages have an effect, which would require sampling of larvae each day after inoculation. However, *Pseudomonas* densities are highly heterogeneous over time in any single tissue [8], so such assays may need to destructively sample the entire worm instead of just hemolymph (and could thus be done equally well with waxworms or other small host species). It would also be interesting to evaluate treatment outcomes at lower temperatures than here since rearing temperature can affect duration of larval stages both in waxworms and hornworms [33,34,35,36]. Worms in our study were reared at 28 °C, and using a lower temperature would increase the length of instars and might equally extend the duration of infection, providing a greater sensitivity of outcome analysis.

This study is both an evaluation of phage therapy and an evaluation of an insect host that might be used more extensively for phage therapy (hornworms). Waxworms (the larvae of a small moth species that parasitizes honeybee hives) have more commonly been used than hornworms as models of bacterial infection [6,7,29,30,33]. Hornworms offer both advantages and disadvantages over waxworms. The main advantage is that hornworms are large, beginning their fifth instar between 1 and 2 g and ending at approximately 10 g [8]. The large size enables easy handling, monitoring of weight and even sequential sampling of hemolymph (the latter not used here). Waxworms are less than a tenth of this weight [6,34]. The individual rearing of hornworms also allows for direct monitoring of the fifth instar onset, providing easy standardization of treatment with developmental stage.

A major disadvantage of hornworms relative to waxworms is that hornworms need to be housed individually from hatching onward, due to cannibalism [8]. This drawback is not biological but is one of time and effort required to conduct a study. However, given our discovery of occasional phage contamination of hornworms, a suitable precaution with any host species would be to house them individually after treatment to prevent phage transfer, thus adding a layer of effort for waxworms approaching that of hornworms. A biological problem with hornworms is that they ingest frass. Our daily inspection of hornworms included the removal of frass, but this was not often enough to fully eliminate frass ingestion. For worms with PAO1 injections, ingestion of frass sometimes caused infection and visible degradation of mouthparts, leading to impaired feeding.

Further analyses beyond those done here or done thus far in waxworms could take the analysis of infections to new levels. In particular, the within-host spatial and temporal dynamics of both bacteria and phage should provide insight into the nature of infection growth and control. The large size of waxworms will enable macroscopic-scale analyses, but such assays could be done on a microscopic scale with small hosts [37].

The ultimate goal here is to identify principles of phage therapy that may be useful in treating infections of humans and domestic animals. The short-term goal was to evaluate whether hornworms are potentially useful model hosts in discovering such principles. We acknowledge that these acute bacterial infections of hornworms (and by extension, other insects) differ in many fundamental ways from bacterial infections of humans. The hope is merely that hornworms may be a useful first step in testing principles, but any suggestion based on hornworms (or other insects) obviously needs to be vetted with more realistic models of humans.

## 4. Methods

### 4.1. Hornworm Husbandry

Hornworm eggs were collected from adult six spotted hawkmoths housed in our lab or purchased from Great Lakes Hornworms (Bruce Township, MI, USA). Eggs were kept at 25 °C until hatching, whence the larvae were housed individually in *Drosophila* tubes and kept in a 28 °C incubator. Worms were fed an artificial wheatgerm-based diet from Great Lakes Hornworms. Once injected, worms were housed in 125 mL plastic flasks with cotton tops, fed *ad libitum*, and monitored daily.

### 4.2. Broth and Media

Luria–Bertani broth (LB) was used to grow bacteria and for phage growth (10 g NaCl, 10 g Tryptone, and 5 g Bacto^TM^ yeast extract per L). Plates used LB with 15 g/L Bacto^TM^ Agar for plaque counts, with phage and cells added to 3 mL of top agar before pouring onto the plate (7 g/L Bacto^TM^ agar in LB). Plates were incubated at 37 °C overnight and plaques or colonies counted the following morning. Difco^TM^ *Pseudomonas* isolation agar plates were used for identification and counting of *Pseudomonas* CFU present in pupae. Media was obtained from Fisher Scientific (Waltham, MA, USA) and Sigma Aldrich (St. Louis, MO, USA).

### 4.3. Bacterial Cells

PAO1 *Pseudomonas aeruginosa* was used for all worm injections. A total of 3 mL of overnight cell culture was added to 5 mL LB in a 125 Erlenmeyer flask and incubated in a shaker at 150 rpm, 37 °C for 2 h to attain log-phase growth. Cells were then centrifuged at 5000 rpm for 3 min, and the pellet was resuspended in 1 mL phosphate-buffered saline (PBS). Cells were further diluted in PBS until a 10^5^ CFU/mL concentration was reached, which was then used promptly for worm injections.

### 4.4. Phage Strains

One of our phages was 99% identical to a *Pseudomonas* phage denoted Φ176, for which we used the same name [38]. SI_6 and SI_7 are novel phages isolated from sewage and were differentiated via plaque morphology followed by sequencing. Sewage isolates were obtained by plating diluted sewage from Austin, Texas, on the PAO1 host. Single plaques were then isolated and grown in LB with the host. High titer phage stocks were prepared by plating roughly 10^6^ phages on PAO1 on LB plates and then incubating overnight. The top agar layer was scraped off and collected, chloroformed and centrifuged. The phage-containing lysate was collected and filter-sterilized with a 0.22 μm syringe filter. Phage titer was confirmed via plating.

### 4.5. Phage Sequencing

Phage lysates were subjected to cesium density centrifugation to recover purified phages. DNA was extracted from purified phages by phenol followed by ethanol or propanol precipitation. Phage genome sequencing and assembly was performed by Plasmidsaurus (South San Francisco, CA, USA) using Oxford nanopore technology with v14 library prep chemistry and R10.4.1 flow cells. Assembled phage genomes were annotated using pharokka v1.9.1 (https://doi.org/10.1093/bioinformatics/btac776) and phold v1.2.3 (https://doi.org/10.1093/nar/gkaf1448). Phages were classified using taxMyPhage v3.3.6 ICTV, MSL41 (https://doi.org/10.1089/phage.2024.0050). Comparative analysis of phage genomes was done using NCBI Blastn from the Blast+ suite v2.17.0 and pairwise alignments in Geneious Prime v2026.0.2.

### 4.6. Hornworm Injections

Injections were performed using a 50 μL Hamilton syringe with a 30-gauge needle. Separate syringes were used for bacterial and phage injections. Each worm was injected with 10 μL of PAO1 bacteria suspended in PBS, aiming for 5 × 10^3^ total CFU. Injections were made on the worm’s side/underside in the crease between the fifth/sixth proleg. After bacterial injection, treatment worms were injected in the opposite side with 10 μL of phage preparation (10^6^–10^8^ PFU).

### 4.7. Daily Monitoring

Worms were weighed daily and food was replenished as needed. Worms were recorded as dead when tactile probing no longer elicited a response. Worms were recorded as pre-pupal when they began body-wetting and developed a pulsing dorsal aorta. All control worms were tested for the presence of phages, and only phage-free controls were included. Occasional phage contamination arose because the worms are necessarily raised in non-sterile conditions, and the handling of vials and frass of worms treated with phage resulted in phage crossover. All control worms were monitored at death for the presence of phage by plating a sample of their internal contents.

### 4.8. CFU Assessment in Pupae

Pupa from PAO1 controls and phage treatments were tested for *Pseudomonas* levels. Pupae were transected via a circumferential incision at the midline. A 10 μL liquid sample was then pipetted onto Difco^TM^ *Pseudomonas* isolation agar plates followed by 48 h incubation at 37 °C. *Pseudomonas* colonies are identifiable by green coloration of the media. Samples were extracted from brown fluid present in the innermost section of the pupae while avoiding more structured support tissues present around the periphery of the cocoon. It should be noted that bacteria may have been sequestered in specific regions of each individual pupae that were not accessed during sampling.

### 4.9. Statistical Analysis

Tests of heterogeneity in survival used Chi-squared tests of 2 × N tables; tests of growth data used standard analysis of variance. Credible intervals for the survival data given in the figures used the Jeffreys interval, the standard Bayesian default for binomial credible intervals [39].

## Figures and Tables

**Table 1:** Hornworm outcomes by treatment. All worms (except PBS controls) were injected with PAO1 *Pseudomonas aeruginosa*, with doses in the range of 10–7.7 × 10^3^ CFU. In worms receiving doses of ~10^3^ CFU of PAO1, three different phages were tested, two novel phages isolated from sewage (SI_6, SI_7) and *Pseudomonas* phage Φ176. Numbers of phages injected were varied to assess effects on survival and weight per phage dose. Average worm weights per treatment group are shown with one standard error in parentheses. Average weights include only surviving worms. Days to pupation ranged from 5 to 9 days post injection. CFU is the number of colony forming units, PFU is the number of plaque forming units, and “n” is the sample size. Worms alive at the time of entry into pupation were considered to have survived, typically by day 7 post injection but sometimes as late as day 9.

Treatment	CFU Administered	PFU Administered	n	% Survival	Avg. Weight (Day 4)	Avg. Weight (Pupation)
PBS Control	-	-	9	100	9.71 g (0.30)	9.71 g (0.30)
PAO1 only	10	-	16	73.3	8.67 g (0.48)	9.18 g (0.55)
PAO1 only	1.5 × 10^2^	-	21	39.1	8.10 g (0.82)	9.38 g (0.33)
PAO1 only	1.2 × 10^3^–7.7 × 10^3^	-	45	17.8	6.25 g (0.31)	8.57 g (0.44)
SI_6	6.9 × 10^3^	7 × 10^6^	25	28.0	6.88 g (0.52)	9.96 g (0.32)
SI_6	7 × 10^3^	4 × 10^7^	25	60.0	7.14 g (0.42)	8.45 g (0.16)
SI_6	2 × 10^3^	9 × 10^7^–1 × 10^8^	20	35.0	6.42 g (0.54)	8.71 g (0.20)
SI_6	6.5 × 10^3^	9 × 10^8^–1 × 10^9^	23	47.8	6.63 g (0.51)	8.86 g (0.19)
SI_7	6 × 10^3^	4 × 10^8^	24	62.5	7.74 g (0.38)	9.33 g (0.40)
Phage Φ176	7.7 × 10^3^	5 × 10^7^	10	60.0	7.30 g (0.27)	8.17 g (0.44)

**Table 2:** The predicted, initial phage killing of bacteria depends heavily on injected phage density per mL (P) and the adsorption rate constant (k). Phage densities span the approximate range applied here. Killing/h is the fraction of bacteria infected per hour assuming no change in phage density. Data were generated from Equation (2).

P	k	Killing/h
10^7^	10^−9^	0.45
10^7^	10^−10^	0.06
10^8^	10^−9^	0.998
10^8^	10^−10^	0.45
10^9^	10^−9^	1.0
10^9^	10^−10^	0.998

**Table 3:** CFU counts and pre-pupal weight of dissected pupae. A total of 10 μL from each pupa were plated on *Pseudomonas* identification plates. Two of the three PAO1-only control pupae tested were found to have roughly 5 × 10^4^ *Pseudomonas* CFU/mL, while the single remaining PAO1 control worm nor any of the six phage-treated worms had detectible *Pseudomonas*.

Treatment	Approximate PA01 CFU/mL	Weight at Pupation
PA01-Control	5 × 10^4^	7.45 g
PA01-Control	4.7 × 10^4^	9.96 g
PA01-Control	None detected	8.32 g
SI_6 (4 × 10^7^)	None detected	8.40 g
SI_6 (1 × 10^9^)	None detected	6.59 g
SI_6 (1 × 10^8^)	None detected	7.38 g
SI_7 (4 × 10^8^)	None detected	9.82 g
SI_7 (4 × 10^8^)	None detected	9.60 g
Phage Φ176 (5 × 10^7^)	None detected	7.60 g

## Data Availability

The genomes of sewage isolate phages SI_6 (Luzseptimavirus—accession number PZ531171) and SI_7 (Pbunavirus—accession number PZ531172) used in this study have been submitted to NCBI. The few differences in the sequence of our Φ176 relative to a published sequence are described in the text. Hornworm size and survival data are pro-vided in the figures and tables.
